# The Special Meeting of the B.M.A.

**Published:** 1919-04-19

**Authors:** 


### The Hospital

The Workers' Newspaper of Administrative Medicine and Institutional Life, Administration, National Insurance and Health.

No. 1715, Vol. LXVI. SATURDAY, APRIL 19, 1919. PRICE TWOPENCE

### THE SPECIAL MEETING OF THE B.M.A.

The governing authorities of the British Medical Association have long been accustomed to hear the voice of criticism relative to their medico-political activities, and it cannot be said that in this respect they have received much support from the recognised leaders of the profession. But their decision to hold a special meeting for the purpose of discussing the medical and surgical lessons of the wars, and to do this while numbers of our professional *confrères* from the United States and from the Dominions were still with us, evoked a positive chorus of approval, and the result has shown that whatever may be true in reference to medical politics, the Association on its clinical side is able to engage the active sympathy and support of every one who counts in the world of medicine. We offer our own congratulations both on the sound judgment which showed itself ready to recognise opportunity and on the efficient and successful manner in which the scheme has been carried into action. The occasion will have a distinct place in the history of the Association, and the enterprise and its realisation are proofs of a vitality which may confidently be expected still further to serve the interests alike of the profession and of the public.

Perhaps the first remark which may be made on a general review of the proceedings of the special meeting is one of compliment to the doctors who have been actively engaged in the war on the zeal and industry with which they have utilised the opportunities placed in their hands, and this in not a few cases in circumstances hardly provocative of scientific accuracy or enthusiasm. Even in or close to the fighting line good work has been done, not merely in the succour of individual patients, but in the acquisition and registration of practical lessons and serviceable knowledge. In some sheltered situations the accumulation of large numbers of patients has offered special opportunities to the medical officers in charge, and these have not been slow to avail themselves of such exceptional occasions. The number of positive contributions to medical and surgical knowledge made by officers engaged on more or less active service has in short been one of the features of the war services of the profession, and many of these have reached a high order of excellence. The B.M.A. meeting of last week gave an opportunity for bringing many of these contributions under a general review, and the resulting discussions were in not a few instances both keen and instructive. The medical profession has taken its full share in the national struggle, and its ranks have been thinned both by wounds and by sickness. Yet amidst all difficulties its members have not failed in their traditional obligation to enlarge the bounds of useful knowledge and to try by the discovery of truth and the acquisition of skill to minister more efficiently to the "relief of man's estate."

A second impression made by a perusal of these professional discussions is a realisation of the extraordinary complexity of the problems with which modern medicine is confronted. The struggle continually is to get behind the superficial and obvious phenomena of disease to the underlying agencies and causes, and this demand is presented in the case of the individual sufferer as well as by the questions grouped under the heading of preventive medicine. To bring to the bedside of the patient, whether for purposes of diagnosis or for those of treatment, the most modern knowledge is becoming a burden which the practitioner is going to find it increasingly difficult to carry. Certain clinical investigations and tests universally recognised as essential to accurate diagnosis are already outside the time and skill of the clinician, and are left necessarily in the hands of the laboratory worker; and the number of these is likely to increase rather than diminish. It is the fashion for the moment to exalt what is called "team work" in medicine, and the thing itself, whatever it may be named, has its value, and indeed may present a plea of necessity. All the same, there must be in the care of a patient, as in the sailing of a ship, some final and co-ordinating authority. Unhappy indeed is the fate of a sick man who is reported on in this, that, and the other respect by a succession of "specialists," but who has no general and overruling direction from a practitioner of wide views and sound judgment. Whatever be the disease and whatever the offending organ there always exists the man and the patient, and this problem is not resolved by a statement of the measure of each of his visceral activities. By all means let us have efficient officers in each of the several departments, but not less necessary is the commander on the bridge if a consistent and successful course is to be pursued. The "team" has its place and value, but its members need a voice to keep each of them in his appropriate place. In preventive medicine this is realised, and is largely carried into practice in our municipal health organisations. A similar principle has to be applied in curative medicine, and an immediate issue for all concerned in the future of the profession is the devising of an educational scheme by which the family practitioner can be fitted for this highly responsible function.

To the discussion of the issue just stated the President of the recent meeting, Sir Clifford Allbutt, devotes a portion of his introductory address, and the contribution is a noteworthy one and equal—no higher compliment is possible—to the reputation of the speaker. The clinician and the laboratory worker must be kept in touch with one another if scientific advances are to be turned to beneficial ends in medical practice. This can only be done through an agency capable of appreciating both points of view. In short, the teachers in our medical schools must fit themselves to undertake this task, and much of what is now a mere pretence at medical education must be swept away. This utterance will not be welcomed in certain quarters, but personally we are convinced it must be recognised as an essential truth if medicine in this country is to be worthy of its traditions and equal to the future. The Clinical Meeting recorded many interesting and valuable lessons in the several departments of medical knowledge, and in the introductory address is to be found a survey of the whole position, and the guidance there indicated comes from a master-hand.

